# Prolonged exposure to freezing stress reduces the ability of chickpea seedlings to effectively tolerate extremely low temperatures

**DOI:** 10.3389/fpls.2023.1239008

**Published:** 2023-11-23

**Authors:** Jafar Nabati, Ahmad Nezami, Alireza Hasanfard, Zahra Nemati, Nastaran Kahrom

**Affiliations:** ^1^ Department of Agrotechnology, Faculty of Agriculture, Ferdowsi University of Mashhad, Mashhad, Iran; ^2^ Department of Horticultural Science, Faculty of Agriculture, Ferdowsi University of Mashhad, Mashhad, Iran; ^3^ Department of Range Management, Faculty of Natural Resources and Marine Sciences, Tarbiat Modares University, Noor, Iran

**Keywords:** autumn chickpea, membrane stability index, peroxidase activity, photosystem II, pigment content

## Abstract

The duration and intensity of freezing stress are the most critical factors determining injury in autumn chickpeas, limiting their production and development. To evaluate the effects of freezing temperature and duration on the survival rate (SU%), as well as the physiological and biochemical characteristics of autumn chickpea seedlings, a study was conducted using five different temperatures (0, -6, -8, -10, and -12°C) and five different durations (1 h, 2 h, 3 h, 4 h, and 5 h) of exposure to freezing stress. The SU% of chickpea seedlings decreased to zero after exposure to temperatures of -10°C and -12°C for 5 hours. As the temperature decreased from -8°C to -12°C and the duration of exposure to freezing stress increased from 1 to 5 hours, the leaf membrane stability index decreased by 33%, 48%, 46%, 57%, and 58%, respectively. The highest and lowest total pigment contents were observed after 1 hour at 0°C and 5 hours at -12°C, respectively. The maximum photochemical efficiency of photosystem II (Fv’/Fm’) was not affected by temperatures as low as -8°C in any of the time treatments during the recovery period. However, this parameter’s value decreased as the freezing stress duration increased. At -12°C, the activity of ascorbate peroxidase, catalase, and peroxidase increased by 44.6%, 38.3%, and 33.0%, respectively, as the duration of stress was increased from 1 hour to 5 hours. A positive and significant correlation was observed between plant dry weight, membrane stability index, photosynthetic pigment content, and Fv’/Fm’ with SU% after exposure to freezing stress. The minimum temperature and the maximum duration of freezing stress tolerance in chickpea seedlings were observed at -12°C for two hours. Our findings confirm that prolonging the freezing duration disrupts the defense mechanisms of chickpea seedlings. Therefore, future studies on breeding chickpeas tolerant to freezing stress should concentrate on attributes strongly correlated with SU%.

## Introduction

The nutritional value of chickpeas (*Cicer arietinum* L.) in terms of vitamins, nutrition, and body health has been recently emphasized often by nutritionists in health and food areas in many countries worldwide (Merga and Haji, 2019). The production of 80% of chickpeas by low-income food-deficit countries reveals the unique role of this product as an alternative source of animal protein (FAOSTAT, 2020). Also, this crop is a vital source of protein, calcium, iron, and phosphorus, and it forms an essential part of the diet of vegetarians. Hence, identifying and eliminating the limiting factors of its cultivation worldwide is one of the most critical research objectives of ICRISAT and ICARDA.

Climate change has increased extreme temperature events, restricting the production and development of many plant species. Freezing stress is one of the crucial constraints limiting plant growth and development worldwide (Hasanfard et al., 2022). The growth response of plants in exposure to freezing stress is affected by physiological, biochemical, and morphological processes. Thus, the change in these attributes leads to different yield and yield-related variables (Liliane and Charles, 2020). Nezami et al. (2022) reported that decreasing the temperature to -12°C led to significant modifications in the survival (SU%) and physiological and biochemical attributes of chickpeas, including the content of photosynthetic pigments, anthocyanin, and water-soluble carbohydrates. Karimzadeh Soureshjani et al. (2022) reported that chickpea genotypes exhibit varying responses at different levels, including molecular (gene expression), biochemical, and physiological modifications, to mitigate freezing damages. The damage resulting from freezing stress can be so severe that it leads to the death of the plant (Gull et al., 2019). Therefore, it is necessary to accurately evaluate the damage caused by freezing stress and the mechanisms involved in the tolerance to freezing stress to predict the SU% of the plant species in winter and its recovery capacity. Breeders will also be able to improve plant tolerance by gene manipulation after determining traits effective in tolerance to freezing stress.

The duration of exposure to freezing stress is also an important factor in the successful wintering of plant species. For example, Parvanova et al. (2004) reported that freezing stress for 24 h resulted in more severe damage in wild-type tobacco compared to 12 h. The B189 and DE15 triticale (× *Triticosecale* Wittmack) seedlings took 11 and 13 days, respectively, to reach 50% mortality when kept at -12°C (Gusta et al., 1997). Nevertheless, if the seedlings were held at -15°C, the time was reduced to 2 and 10 days, respectively. Therefore, a plant species can respond differently to freezing stress depending on the temperature patterns of each year and region because the duration and intensity of freezing are often different for each year and region.

Objective evidence has indicated that the freezing injury of winter chickpeas is affected by extreme freezing temperatures and freezing durations during winter. However, what is the tolerance threshold for chickpea freezing stress? How long can chickpeas tolerate freezing stress? Which morphophysiological and biochemical attributes of chickpea change under freezing stress? Can a single freezing stress tolerance threshold be determined for chickpea seedlings? Does the tolerance threshold vary depending on the duration of stress exposure? The responses to these queries can determine the ideal areas for growing this plant worldwide. Most importantly, there are seldom reports on the interaction effect of the duration and intensity of freezing stress on chickpea seedlings. Hence, this study was conducted to determine the impact of the duration and intensity of freezing stress on chickpea seedlings’ response and to clarify the ambiguities mentioned above.

## Materials and methods

### Study site and growing conditions

The experiments were conducted in November 2021 at Ferdowsi University of Mashhad (FUM), Iran. The study area is located in Northeast Iran, where the climate is arid, with cold and relatively wet winters and hot and dry summers. The mean annual temperature and precipitation are 14.6°C and 250 mm, respectively.

**Figure 1:**
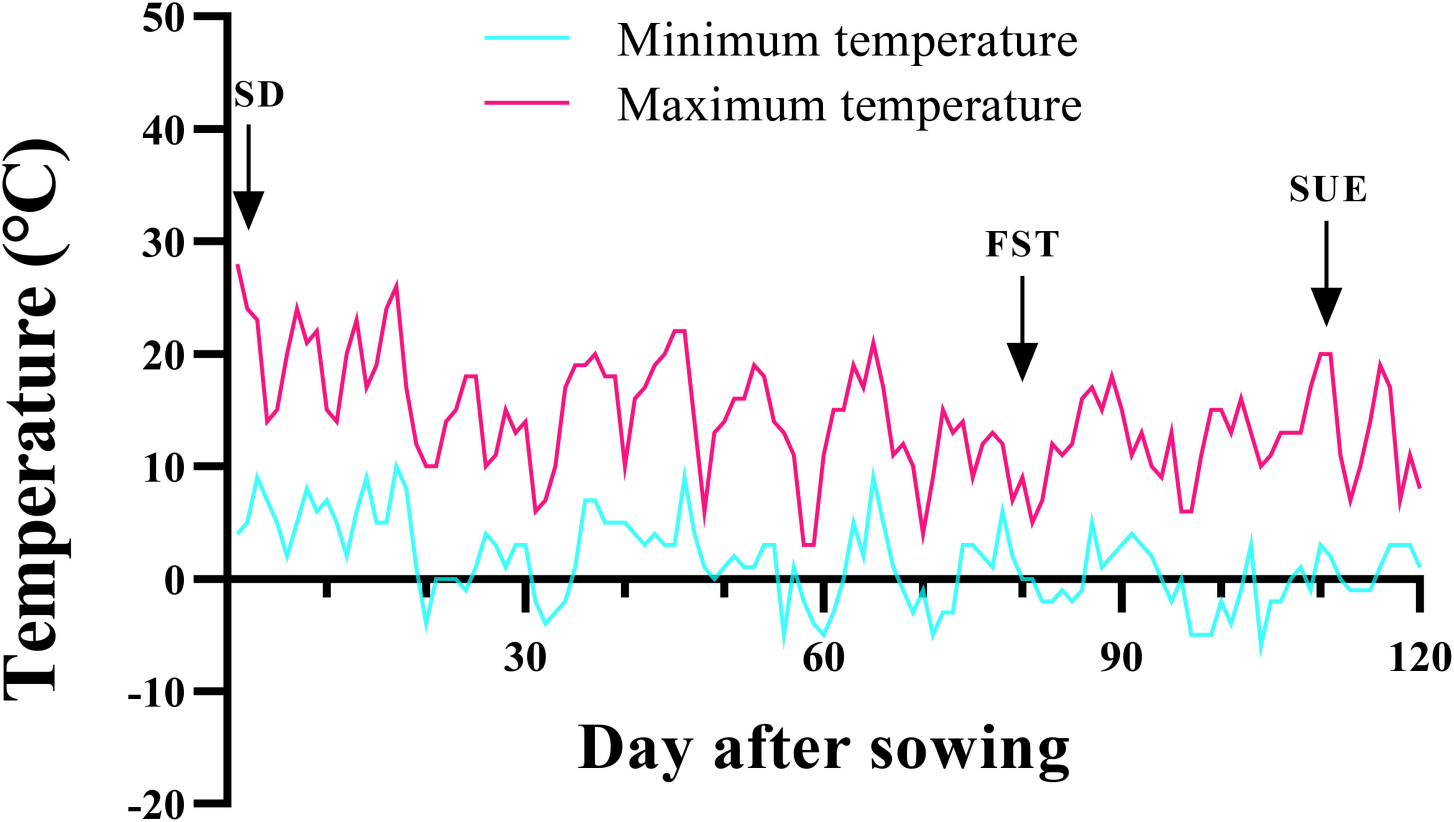
Minimum and maximum daily air temperatures at the experimental site. Relative Humidity ~50% under the natural photoperiod. SD, Sowing date; FST, Freezing stress treatment; SUE, Survival evaluation. The average minimum and maximum long-term temperatures in Mashhad during the period of low temperature application are -5°C and 6°C, respectively.

Seeds were provided from the Mashhad chickpea collection (Research Center for Plant Science) at the FUM. Ten seeds were planted in 14-cm diameter plastic pots filled with sand, soil, and peat moss (1:1:1, v/v). Immediately after emergence, seedlings were thinned to keep eight plants pot^-1^. For cold acclimation, the pots were kept outdoors (**Figure 1**). Pots were regularly watered with potable water. No fertilizer was applied to the crop at any growth stage.

### Duration and intensity of freezing treatment

Chickpea seedlings were exposed to freezing stress at the six-leaf stage. The treatments of this study include freezing temperatures (0, -6, -8, -10, and -12°C) and the duration of exposure of chickpea seedlings to freezing (1, 2, 3, 4, and 5 h). This experiment used a completely randomized design in factorial arrangement (5×5) with four replications.

Freezing treatment was applied using a thermogradient freezer with the ability to adjust the duration and intensity of freezing. The temperature drop slope was adjusted based on the long-term meteorological statistics of Mashhad. The initial temperature of the thermogradient freezer was +5°C. However, the freezer was gradually cooled down at a rate of 2°C per hour. Seedlings were kept at each freezing temperature at the five mentioned times. After the freezing treatment, the pots were transferred to the growth chamber at 5 ± 1°C for approximately 24 hours to slow down the thawing process. Afterward, the pots were transferred to the glasshouse, where they were exposed to natural light conditions, a photoperiod of 16 hours, and a temperature of 16/22°C (day/night) for 30 days. The relative humidity in the glasshouse was maintained at 50% to facilitate recovery.

### Measurement of traits

Freezing stress injury to the cell membrane was measured by electrolyte leakage (EL) using an electrical conductivity meter (Jenway Model 4510, UK). The youngest fully expanded leaves and crown were harvested for the electrolyte leakage assays. The membrane stability index (MSI) was calculated using Eq. 1 and 2.


Eq. 1 EL(%)=(EC1/EC2)×100

Eq. 2 MSI=1−EL%

Where EC1 is the initial conductivity, EC2 is the conductivity of the killed samples (using autoclave 121°C at a pressure of 1.2 atm for 30 min).

Thirty days after freezing treatment, the plants’ SU% in each pot was calculated as SU% = (s_1_/s_2_) × 100. Where s_1_ and s_2_ are the number of alive plants four weeks after freezing treatment and the number of alive plants before freezing treatment, respectively. Plant dry weight was measured after oven-drying at 75°C for 72 h. Three-parameter logistic model (Eq. 3) was used to calculate the lethal temperature for 50% of plants according to the survival (LT_50_ value).


Eq. 3 $$Y=\frac{d}{1+e^{b(\log x-loge)}}$$

Where Y is the response rate (survival %) at temperature x, d is the upper limit for Y, b is the slope of the curve around e, and e is the temperature (°C). Here, e is replaceable with LT_50_.

24 h after each freezing temperature, the youngest fully expanded leaves were harvested for the biochemical assays. All samples were stored at -20°C before being analyzed biochemically. Content of photosynthetic pigments (CPPs) (Dere et al., 1998), anthocyanins (Wagner, 1979), free radical scavenging activity by DPPH (Brand-Williams et al., 1995), flavonoids (Chun et al., 2003), total phenol content (Singleton and Rossi, 1965) and enzymatic antioxidants activity, including ascorbate peroxidase (APX), catalase (CAT), and peroxidase (POD) (Lee and Lee, 2000) were measured by spectrophotometer (Jenway UV-Visible Spectrophotometer Model 6305, UK). Chlorophyll fluorescence parameters (before stress and at 24, 72, and 120 hours after stress) were measured under stable environmental conditions from the youngest fully developed leaf using a pulse-modulated fluorometer (model OS1-FL, Opti-Sciences, Hudson, NH, USA) (Hasanfard et al., 2023a). All measurements were taken on one leaf per plant using sun-exposed leaves under similar environmental conditions between 11:00 and 14:00 on sunny days (Leakey et al., 2006; Li et al., 2019). In this study, Fv’ and Fm’ were used to represent the variable and maximum fluorescence of light-adapted leaves, respectively (Baker, 2008). The leaf osmotic potential was measured using an osmometer (OM802-D; Vogel, Germany).

### Statistical analysis

Data were analyzed using the SAS 9.4 software (*v.* 9.4, SAS Institute Inc, Cary, NC, USA) and means were compared using Fisher’s LSD (least significant difference) test at a 5% probability level. GraphPad Prism software (*v.* 8.00; GraphPad, CA, USA) was used to draw the graphs.

## Results

### Survival (SU%) and dry weight

**Figure 2:**
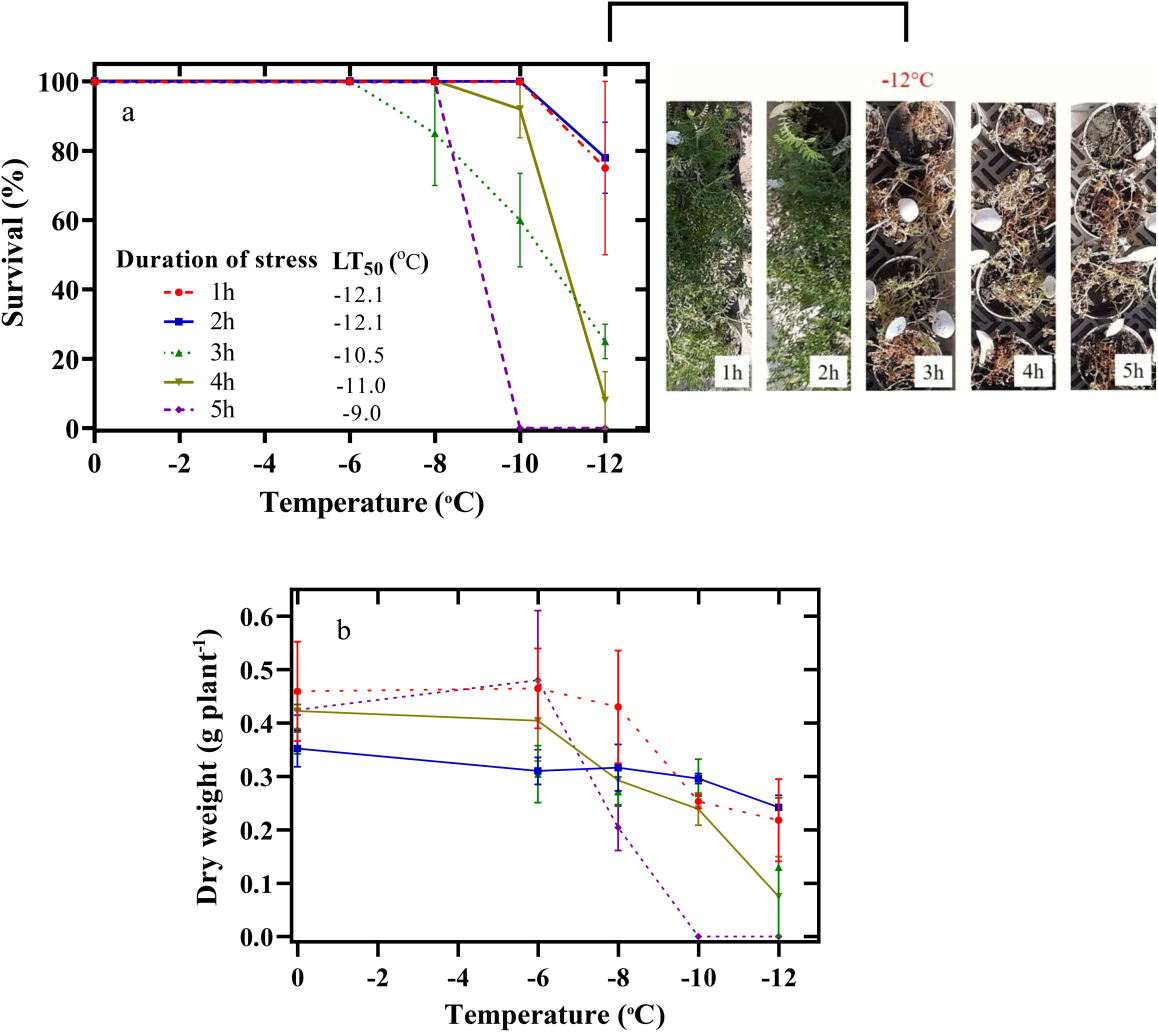
Effect of freezing temperatures on the survival percentage **(A)** and dry weight of the aerial parts **(B)** in chickpeas. Vertical bars indicate the standard error of means.

The lowest SU% of chickpea seedlings was observed at -12°C (**Figure 2A**). Thus, chickpea seedlings were exposed to -12°C for one to 5 h, and their SU decreased to 75%, 78%, 25%, 8%, and 0%, respectively. The estimated LT_50_ was -12.1, -12.1, -10.5, -11, and -9°C during the freezing stress periods of 1, 2, 3, 4, and 5 hours, respectively (**Figure 2A**).

A significant decrease in the dry weight of the plant began during the 5-h treatment at -6°C (**Figure 2B**). In this way, the dry weight of the plant decreased by 51% at -8°C and by 100% at -10 and -12°C. The lowest dry weight of the plant in other periods of freezing stress (1 h to 5 h) was also observed at -12°C.

### Physiological and biochemical attributes

#### The membrane stability index

**Figure 3:**
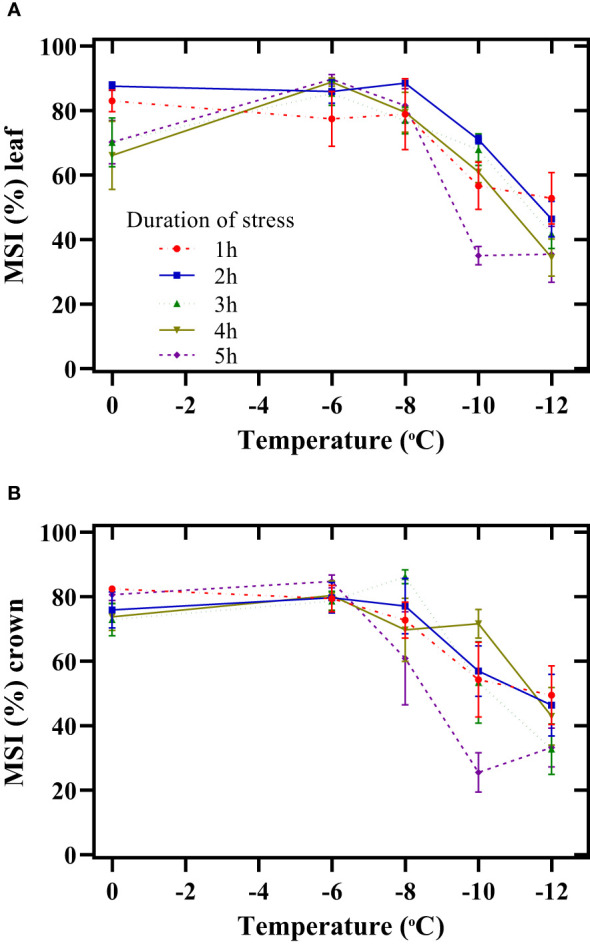
Effect of freezing temperatures on the membrane stability index (MSI) of the leaf **(A)** and crown **(B)** in chickpeas. Vertical bars indicate the standard error of means.

The maximum decrease in the MSI of the leaf during the stress period began at -8°C (**Figure 3A**). Thus, by decreasing the temperature from -8 to -12°C and increasing the duration of exposure to freezing stress from 1 h to 5 h, the MSI of the leaf decreased by 33%, 48%, 46%, 57%, and 58%, respectively. In all stress periods, the crown membrane’s lowest stability index was observed at -12°C (**Figure 3B**). The decrease in this index was more intense when exposed to stress for 5 h. Thus, exposing the seedlings for 5 h at -12°C decreased the MSI of the crown by 61% compared to -6°C.

#### Content of photosynthetic pigments

**Figure 4:**
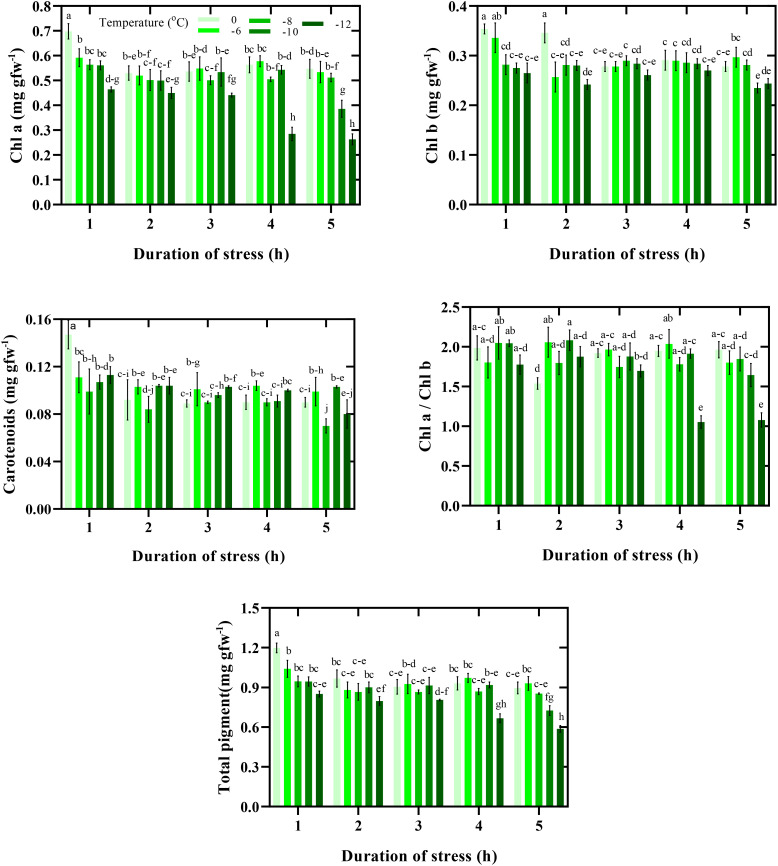
Content of photosynthetic pigments response to duration and intensity of freezing stress. Vertical bars indicate the standard error of means, and bars with the same letter indicate no significant differences according to LSD at P ≤ 0.05.

Duration and intensity of freezing stress significantly affected CPPs (**Figure 4**). The highest CPPs were observed in the control conditions (0°C for 1 h). Although freezing stress decreased CPPs, the percentage and decrease trend differed depending on the duration of exposure to stress. The lowest chlorophyll a content was observed when exposed to -12°C for 4 and 5 h. Thus, the chlorophyll a content in the mentioned conditions was 61% lower than in the control. In general, a similar trend was observed in other photosynthetic pigments. In this way, with decreasing temperature and increasing the duration of exposure to freezing stress, the CPPs in leaves decreased further. Therefore, there was a 50% difference between the highest total pigment content (0°C for 1 h) and the lowest (-12°C for 5 h) (**Figure 4**).

#### Chlorophyll fluorescence parameters

**Figure 5:**
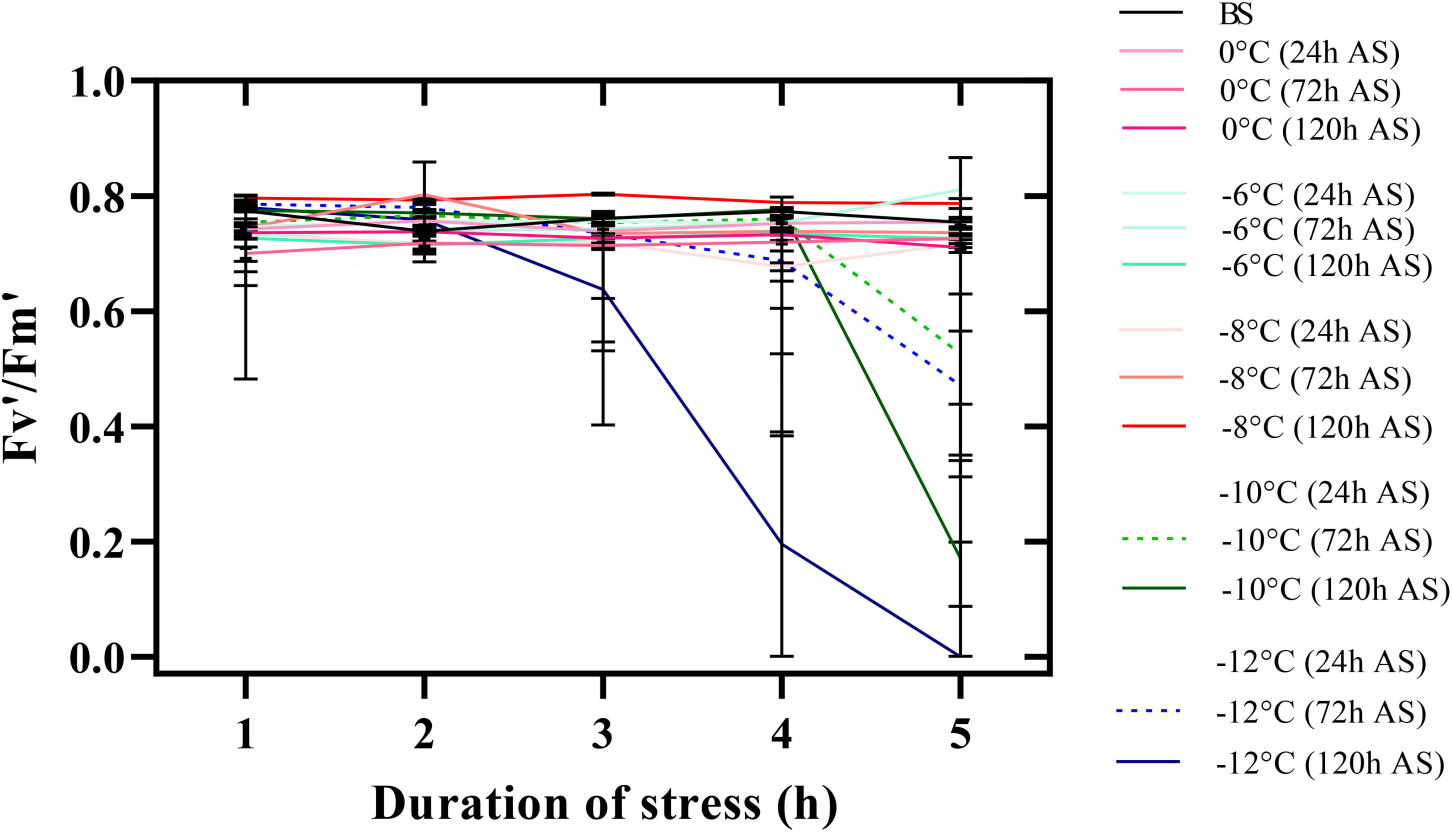
Changes in the maximum photochemical efficiency of photosystem II (Fv’/Fm’) of chickpea seedlings under freezing temperature and duration in the recovery period. The error bars indicate standard error. BS (before stress) and AS (after stress).

A significant decrease in the maximum photochemical efficiency of photosystem II (Fv’/Fm’) was clear from -10°C. The lowest Fv’/Fm’ was observed in all temperature treatments under stress for 5 h (**Figure 5**). Thus, Fv’/Fm’ in seedlings reached zero 120 h after freezing stress (-12°C for 5 h). By decreasing the temperature to -8°C, Fv’/Fm’ was not affected in any of the time treatments in the recovery period. With increasing exposure time to freezing stress, other chlorophyll fluorescence parameters (Fq’/Fm’, Fq’/Fv,’ and qL’) also decreased (**Supplementary Table S1**). Generally, the parameters’ lowest values were observed when chickpea seedlings were exposed to -12°C for 5 h.

#### Anthocyanins

The highest anthocyanin content in each temperature treatment was observed when the seedlings were exposed to freezing stress for 5 h (**Table 1**). In other words, the content of anthocyanin increased at each temperature with increasing duration of exposure to stress. The anthocyanin content increased by 23%, 20%, 29%, and 38%, respectively, when exposed to -6, -8, -10, and -12°C for 5 h compared to the control (stress for 1 h).

**Table 1:** Changes in the anthocyanins (ACNs), free radical scavenging activity (DPPH), flavonoid content, phenol content, and leaf osmotic potential (OP) of chickpea seedlings under freezing temperature and duration.

Temperature (°C)	Time (h)	ACNs^⸸^ (mmol.gfw^-1^)	SE^⸸⸸^ (±)	DPPH (mg.gfw^-1^)	SE (±)	Flavonoids (mg.gfw^-1^)	SE (±)	Phenol (mg.gfw^-1^)	SE (±)	|OP| (Mpa)	SE (±)
0	1	0.250d-h	0.006	3.729a-c	0.533	14.073a	0.391	213b-e	2.731	2.974a	0.002
0	2	0.261c-g	0.008	3.103b-f	0.028	11.110b-d	0.366	183i-k	2.469	2.692a-e	0.141
0	3	0.268b-f	0.004	2.764d-g	0.315	11.818b	1.026	185h-k	2.439	2.273d-h	0.172
0	4	0.245e-h	0.009	3.357a-f	0.371	11.357bc	0.774	187g-k	2.685	2.608a-f	0.153
0	5	0.276b-e	0.010	3.958ab	0.214	11.233bc	0.536	191f-k	4.290	2.453b-g	0.101
-6	1	0.223h-k	0.009	2.664d-g	0.315	9.514d-j	0.437	201d-i	8.424	2.563a-f	0.208
-6	2	0.246e-h	0.014	2.702d-g	0.195	9.410d-j	0.805	181jk	2.630	2.054g-j	0.259
-6	3	0.244f-h	0.020	2.608e-g	0.454	8.996g-j	0.788	187h-k	5.853	2.491a-g	0.243
-6	4	0.244f-h	0.009	3.166a-f	0.271	9.009g-j	0.639	184h-k	7.682	2.200f-h	0.062
-6	5	0.291a-c	0.006	3.403a-e	0.260	9.992c-i	0.701	199e-j	15.388	1.868h-j	0.145
-8	1	0.235g-j	0.006	3.996a	0.508	10.940b-e	0.566	218a-d	9.132	2.761a-d	0.143
-8	2	0.241f-i	0.019	3.420a-e	0.273	9.185f-j	0.351	197e-j	6.035	2.384c-g	0.172
-8	3	0.221h-k	0.007	3.193a-f	0.153	8.373i-k	0.830	197e-j	6.293	2.912ab	0.180
-8	4	0.231g-j	0.014	3.204a-f	0.321	8.969g-j	0.446	186h-k	9.586	2.252e-h	0.128
-8	5	0.294ab	0.008	2.701d-g	0.161	8.613h-j	0.655	194g-j	1.001	1.835h-j	0.160
-10	1	0.206jk	0.021	2.521fg	0.334	10.343b-g	0.680	212b-e	3.704	2.836a-c	0.126
-10	2	0.235g-j	0.008	3.504a-d	0.182	10.808b-f	0.303	218a-d	1.455	2.921ab	0.131
-10	3	0.239f-i	0.018	3.692a-c	0.223	11.960b	0.527	205c-g	2.299	2.524a-g	0.213
-10	4	0.235g-j	0.007	3.682a-c	0.438	10.253b-h	0.197	203c-h	8.158	2.168f-i	0.321
-10	5	0.292a-c	0.014	2.966c-f	0.194	9.928c-i	0.382	175k	9.675	1.693ij	0.075
-12	1	0.193k	0.010	3.148a-f	0.385	9.463d-j	0.422	187g-k	5.629	2.588a-f	0.192
-12	2	0.226h-j	0.008	3.192a-f	0.018	9.341e-j	0.350	209b-f	0.382	2.057g-j	0.101
-12	3	0.212h-k	0.003	2.890c-g	0.423	8.172j-k	0.888	221a-c	4.655	2.164f-i	0.226
-12	4	0.278b-d	0.011	2.751d-g	0.093	8.315i-k	0.833	225ab	4.209	2.042g-j	0.233
-12	5	0.313a	0.000	2.050g	0.212	6.851k	0.371	233a	7.122	1.623j	0.095

^⸸^In each column, means with similar letters do not have a significant difference in the probability level of 0.05 based on the least significant difference (LSD) test.

^⸸⸸^Standard error.

Lowercase letters mean in each column that similar letters do not have a significant difference at the probability level of 0.05 based on the least significant difference (LSD) test.

#### Free radical scavenging activity

Increasing the duration of freezing stress increased DPPH (**Table 1**). In contrast, DPPH decreased with an increase in the stress period at -12°C. In the most extreme conditions of freezing stress (-12°C for 5 h), DPPH was 45% lower than the control.

#### Flavonoids

The highest flavonoid content was observed in the control (**Table 1**). Generally, the flavonoid content decreased with decreasing temperature and increased exposure duration to freezing compared to the control. In the most extreme conditions of freezing stress (-12°C for 5 h), the flavonoid content was 51% lower than the control.

#### Total phenol content

The trend of increasing the phenol content at -12°C was clear with increasing the duration of stress (**Table 1**). The phenol content increased by 11%, 15%, 17%, and 20% when exposed to -12°C for 2 to 5 h compared to the control (stress for 1 h).

#### Leaf osmotic potential

The osmotic adjustment was almost done by exposing the seedlings to -6 to -12°C for 1 to 4 h (**Table 1**). During the mentioned conditions, the osmotic potential of the leaves of the seedlings was almost in the same range. The highest osmotic potential among the treatments was observed when the seedlings were exposed to -12°C for 5 h. The osmotic potential in these conditions was 37% higher than the control (stress for 1 h).

#### Enzymatic antioxidants activity

**Figure 6:**
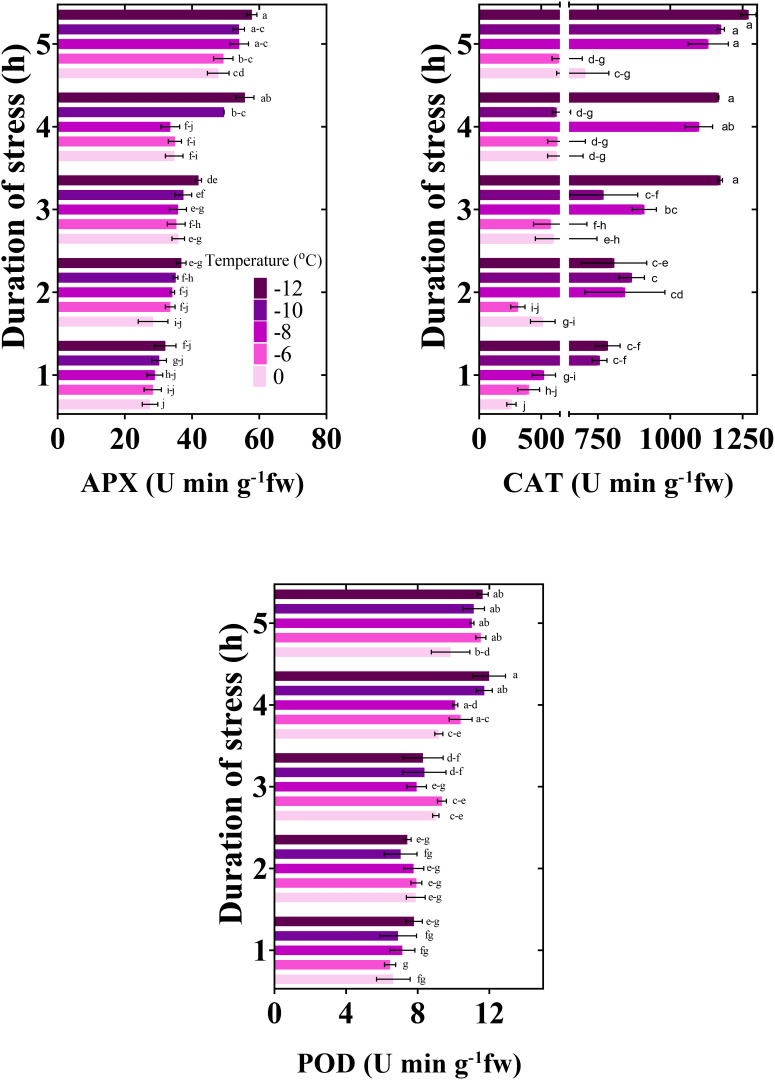
Changes in the antioxidant activity of chickpea seedlings under freezing temperature and duration. APX, ascorbate peroxidase; CAT, catalase; POD, peroxidase. Vertical bars indicate the standard error of means, and bars with the same letter indicate no significant differences according to LSD at P ≤ 0.05.

Significantly, with the decrease in temperature and the increase in the duration of freezing stress, the activity of antioxidants increased (**Figure 6**). The activity of APX increased by 43, 35, 46, 44, and 45%, respectively, when exposed to temperatures from zero to -12°C for 5 h compared to the control (stress for 1 h). CAT activity increased by 63%, 38%, 54%, 36%, and 38%, and POD activity by 33, 44, 35, 5, and 33%, respectively, compared to the control.

### Correlation matrix analysis

**Figure 7:**
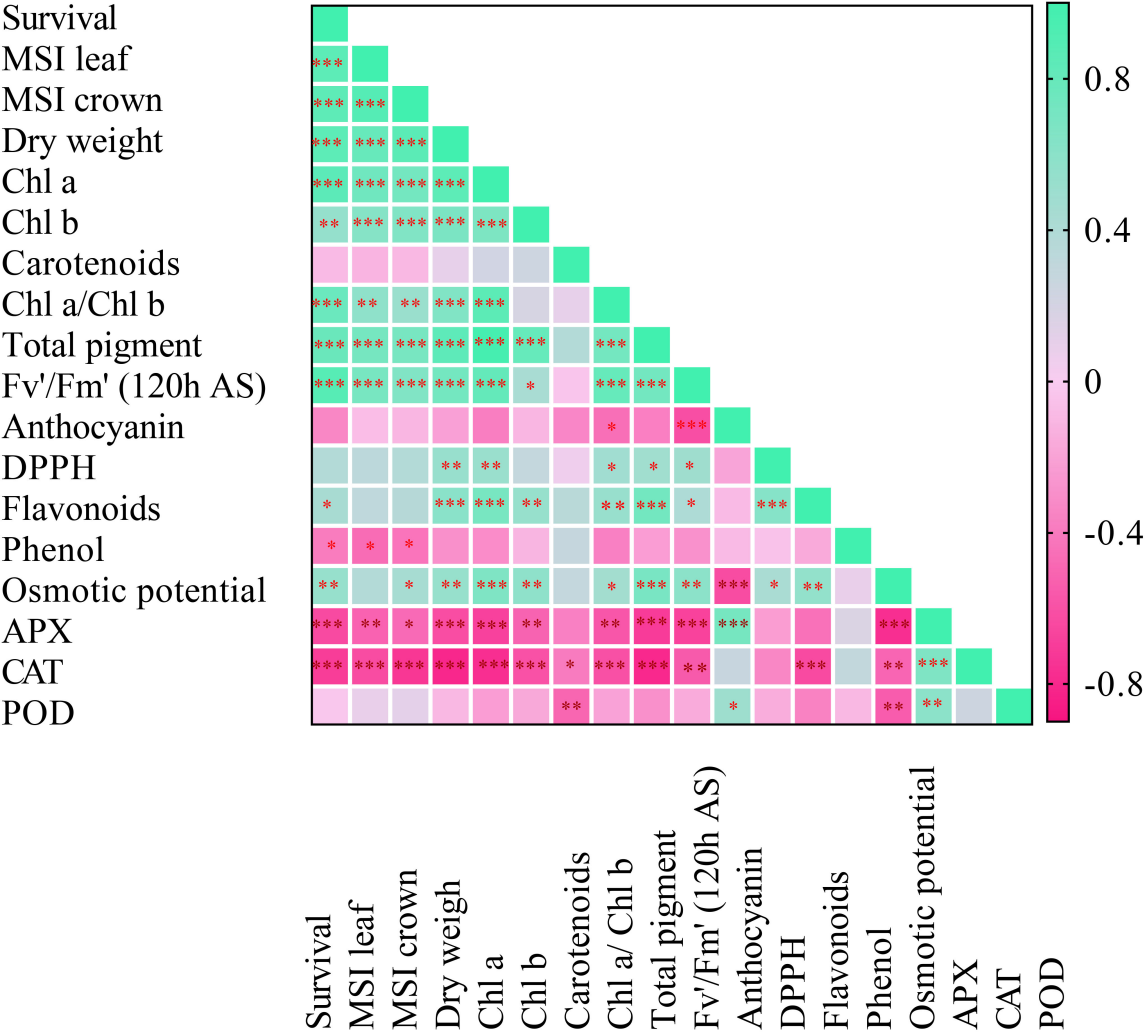
Pearson correlation coefficient matrix between chickpea seedlings’ biochemical, physiological, and morphological attributes under freezing temperature and duration. Asterisks denote significant differences: *p ≤ 0.05; **p ≤ 0.01 and ***p ≤ 0.001.

Pearson’s correlation analysis was performed among chickpea seedlings’ morphological, physiological, and biochemical parameters under freezing stress (**Figure 7**). Under freezing stress, SU% had a positive and significant correlation with MSI% leaf (0.84^***^), MSI% crown (0.84^***^), and plant dry weight (0.85^***^). Between SU% and the content of photosynthetic pigments including Chl a (0.85^***^), Chl b (0.54^***^), Chl a/Chl b (0.77^***^), and total pigment content (0.78^***^), a positive and significant correlation was observed. Further, a significantly stronger correlation was exhibited between SU% and Fv'/Fm' (0.88^***^), while APX (-0.64^***^) and CAT (-0.70^***^) were negatively associated with SU%. Interestingly, MSI% leaf was positively and highly correlated with Fv'/Fm' (0.70^***^); however, APX (-0.52^***^) and CAT (-0.63^***^) had a strong negative correlation with MSI% leaf.

## Discussion

Determining SU% is one of the most common and accurate methods of evaluating plant species’ tolerance to freezing stress. The ability of plant species to successfully overwinter leads to the development of their cultivation (Nezami et al., 2023). Therefore, the expansion of geographical distribution and autumn cultivation of valuable plants such as chickpeas requires screening traits most physiologically and biochemically related to the SU%. In fact, a higher winter SU% ensures the successful cultivation of chickpeas in autumn. The spring season, due to its shorter duration, reduces the productivity of chickpeas compared to longer winter seasons. Autumn cultivation provides more time for crops to grow, resulting in higher yields (Rani et al., 2020). In our study, the SU% of chickpea seedlings decreased with increasing duration of exposure to freezing stress (**Figure 2A**). Thus, with the increase in the duration of exposure to freezing stress, the LT_50_ of chickpea seedlings increased (**Figure 2A**). A similar study on winter cereals revealed that LT_50_ was negatively affected by the length of exposure to -4°C (Willick et al., 2021). Analyzing chickpea seedlings’ response under freezing stress showed that their maximum capacity (SU=100%) was at -8°C for 5 h. The chickpea plants maintained a high survival rate even under unfavorable conditions, as they were able to withstand exposure to -12°C for two hours (**Figure 2A**). The exposure of chickpeas to -10 and -12°C for 5 h led to their mortality (SU=0%).

The increase in the intensity and duration of freezing stress is effective on morphological attributes, including the accumulation of dry matter. In our study, dry matter accumulation decreased under increasing freezing stress duration. The ability to accumulate dry matter during the recovery period indicates the activity of the plant’s photosynthetic apparatus. Nabati et al. (2021) showed that a decrease in temperature from 0°C to -4°C caused a 20% decrease in the dry weight of the aerial parts of faba bean (*Vicia faba* L.) seedlings as a result of a disturbance in gas exchange variables.

Understanding the physiological and biochemical attributes effective in increasing tolerance to freezing stress and breeding chickpeas based on these factors will increase the cultivation of this plant worldwide. The plasma membrane is the primary target of freezing stress, affecting its conformation and properties and disturbing cellular homeostasis. Degradation of cellular membranes is commonly observed following freeze-thaw stress to plant tissue. Therefore, any disturbance in the membrane structure causes severe damage to the plant. Our objective evidence from various studies has shown that the destruction of the crown of chickpea seedlings is highly likely to result in their mortality. However, there is a high possibility of regrowth of leaves after freezing stress, and the plant can recover. This mindset was aligned with the findings of the study. In this study, the reduction of MSI (leaf and crown) increased with a decrease in temperature and an increasing duration of exposure to freezing stress (**Figure 3A, B**). Increasing the duration of exposure to freezing stress (-12°C) probably changed the membrane structure in terms of the quantity and quality of fatty acids and proteins. Subsequently, the plant growth regulation and mediating responses to stress were affected. Willick et al. (2021) reported that due to global climate change in Western Canada, winter cereals would be exposed to a higher frequency of winter thaws, increasing crown water and reducing freezing tolerance.

Photosynthetic pigments participating in the absorption, transformation, and transfer of light energy play a crucial role in plant growth (Hasanfard et al., 2023b). Under freezing stress, lipid peroxidation and reactive oxygen species (ROS) are important in damaging photosynthetic membranes. The results of our study confirmed that the CPPs could be an advantageous screening technique to investigate the tolerance to freezing in chickpea seedlings (**Figure 4**). Improvement in pigment content and photosystem functions in plant species increases photosynthetic efficiency. In this way, breeders can concentrate on CPPs to decrease the effects of freezing stress. For example, An et al. (2020) reported that using some modifiers can reduce damage to CPPs and therefore decrease the effects of stress in plants.

The sensitivity of photosystem II activity to freezing stress has made chlorophyll fluorescence analysis a practical technique for understanding photosynthetic mechanisms. Our study revealed that chlorophyll’s fluorescence parameters could be suitable criteria for determining the activity of the plant’s photosynthetic apparatus (**Figure 5**). Freezing stress decreased the quantum yield of the electron transport flux from QA to QB of photosystem II. The longer chickpea seedlings were exposed to low temperatures, the more their recovery ability decreased. Increasing the duration of exposure to freezing stress led to a decrease in the amount of electron transfer to electron acceptors of photosystem II and disruption of the activity of the photosystem II electron transfer chain. Therefore, the ability to recover and the high performance of the photosynthetic apparatus of a species after exposure to freezing stress indicate the high adaptability of that species to harsh winter conditions.

Contrary to our assumption, flavonoid content did not increase with increasing freezing stress. This result showed that all biochemical attributes in plant species do not primarily function in stress tolerance. Hence, according to the results of this study, we could not prove the role of flavonoids in tolerance to freezing stress, and we propose it as an ambiguity.

Anthocyanins are a class of water-soluble flavonoids that have many biological functions in higher plants. To adapt to freezing stress, plants have adaptive responses, including the accumulation of anthocyanins. Here we demonstrated that freezing stress-induced anthocyanins contribute substantially to the low-temperature tolerance of chickpea seedlings (**Table 1**). We also concluded that anthocyanin accumulation is ineffective in plant tolerance if freezing stress is prolonged.

The increase in free radicals and, as a result, cell destruction has caused “oxidative stress.” This phenomenon destroys proteins and lipids. The increase in DPPH in conditions of severe freezing stress indicates the increase in free radicals. In the most severe freezing stress treatment, the increase of DPPH has stopped, so inhibition of free radical activity by DPPH as a single defense mechanism against freezing stress in chickpea seedlings is impossible. With the increase in reactive oxygen species, various mechanisms are activated in plants. Our study determined that the activity of antioxidant enzymes (APX, CAT, and POD) increased with exposure to freezing stress (**Figure 6**). In other words, ROS-scavenging enzymes have increased to decrease the toxic effects caused by oxidative stress resulting from freezing stress. Freezing stress can cause damage to plant cells through the formation of ice crystals, which can disrupt cellular structures and membranes. ROS production increases during freezing stress due to the disruption of cellular homeostasis. However, plants have evolved mechanisms to counteract the harmful effects of ROS and mitigate the damage caused by freezing stress. One of the main ways in which ROS helps reduce the effects of freezing stress is through its involvement in signaling pathways that activate various defense mechanisms. ROS can act as signaling molecules to trigger the expression of genes involved in stress responses, including the production of protective proteins and antioxidants. In this study, it is likely that these defense mechanisms help chickpea plants maintain cellular integrity and minimize damage caused by freezing stress.

Phenolic compounds play their antioxidant function with several mechanisms, such as free radical scavenging activity and interrupting oxidation chain reactions, donating hydrogen, and acting as a substrate for peroxidase enzymes. In this study, the role of these compounds was observed at -12°C with increasing stress intensity. The phenol content had almost the same trend at different temperatures during the freezing stress period.

Plants could often adjust the reduced potential without water loss, except when the freezing was very severe. The exposure of plants to freezing stress for 5 h disturbed the osmotic adjustment. In other words, the mechanism to prevent water loss (the process of maintaining cellular turgescence) decreased with the increased exposure to stress (stress for 5 h).

The damage of cells under freezing stress depends on the production of reactive oxygen species and the efficiency of detoxification mechanisms in plants. To deal with the created oxidative stress, plants have a highly efficient antioxidant defense system that can neutralize or scavenge free radicals. In this study, the antioxidant enzymes likely significantly reduced the levels of superoxide and hydrogen peroxide in chickpea seedlings. The increase in the levels of antioxidants was more evident at -10 and -12°C and during the stress periods of 4 and 5 h. It can be concluded that the protective function of antioxidant enzymes increases with increasing intensity and duration of freezing stress.

The positive and significant correlation between SU% and MSI% showed that chickpea seedlings could withstand freezing stress with more cell membrane integrity. Plant cell membranes are critical sites for the perception of abiotic stress factors; therefore, plant breeders should endeavor to target these sites in the breeding of plants to tolerate freezing stress. Membrane lipids changed drastically in plants when they suffered from freezing stress. Campos et al. (2003) reported that electrolyte leakage and lipid degradation account for cold sensitivity in the leaves of plants. Freezing stress decreases the contents of total lipids and stimulates lipid degradation, accumulating free fatty acids. In previous studies, researchers reported that the more successful the plant species were in maintaining the integrity of their cell membranes, the fewer electrolytes leaked from their cell membranes, and their SU was higher (Nabati et al., 2020; Angmo et al., 2022).

Photosynthetic pigments are essential in all parts of the photosynthesis system because their quantity and quality play vital roles in plant assimilation (Sayyad-Amin et al., 2016). The positive and significant correlation of photosynthetic pigments with SU% reveals their crucial function in tolerance to freezing stress. When the intensity and duration of freezing stress increased, CPPs in chickpea seedlings decreased significantly (**Figure 4**). In other words, the plant’s photosynthetic apparatus has been disturbed, and the ability of CO_2_ assimilation has decreased. These interactions resulted in the instability of the photosystem and fluorescence parameters of Chl a and the decrease of Rubisco carboxylation efficiency, which led to the decrease of SU% of chickpea seedlings.

Fv’/Fm’ indicates the maximum quantum efficiency of photosystem II photochemistry and is used for early stress detection in plants. Its high and positive correlation with the SU% in this study showed that this parameter could be considered as one of the most reliable techniques for detecting damage to photosynthetic apparatus and tolerance to freezing stress in plants. The high Fv’/Fm’ values in exposure to freezing stress reveal that the electron transfer chain is established in the thylakoid membrane, and the plant can recover adequately after the stress.

Contrary to our expectation, the correlation between SU% and antioxidant enzymes was negative (**Figure 7**). Antioxidant enzymes are plants’ most important defense mechanisms in exposure to stress. This negative association is only due to a severe decrease in SU% and a severe increase in the activity of antioxidants at low temperatures. In this study, the increase in the activity of antioxidants was significant in the most severe freezing treatment. However, this defense mechanism did not lead to the SU of chickpea seedlings.

## Conclusions

The duration of freezing stress was influential in determining the SU of chickpea seedlings, so increasing the stress time (-12°C) from 1 to 5 h led to a decrease in SU% from 75 to zero. The results of our study prove that the interactions of coping with freezing stress in chickpea seedlings are complex. Therefore, a combination of defense mechanisms (morphological, physiological, and biochemical) in plants leads to high tolerance to exposure to freezing stress. Although chickpea seedlings with high antioxidant potential could survive and sustain their growth under freezing stress, this ability was insufficient to cope with freezing stress. Cell membrane stability, CPPs, and the maximum quantum efficiency of photosystem II were effective characteristics in tolerance to freezing stress in chickpea seedlings. Accordingly, chickpea breeders should concentrate on the mentioned attributes in breeding for freezing stress tolerance.

## Data availability statement

The original contributions presented in the study are included in the article/**Supplementary Files**, further inquiries can be directed to the corresponding author.

## Author contributions

JN: Methodology, Reviewing, Editing. AN: Conceptualization, Supervision. AH: Conceptualization, Methodology, Laboratory work, Data collection, Data analysis, Writing. ZN: Laboratory work, Data collection. NK: Laboratory work, Data collection. All authors contributed to the article and approved the submitted version.
