# Supplementary material for: Prolonged exposure to freezing stress reduces the ability of chickpea seedlings to effectively tolerate extremely low temperatures
Source: Front Plant Sci. 2023 Nov 23;14:1239008. doi: 10.3389/fpls.2023.1239008 (PMC10701537; doi:10.3389/fpls.2023.1239008)
Supplement: Supplementary file 1 [file Table_1.docx]

**Supplementary**

**Table 1S** Changes in the photosystem II **(**PSII**)**operating efficiency (Fq′/Fm′), photochemical quenching (Fq′/Fv′), and estimates the fraction of open PSII centers (qL´) of chickpea seedlings under freezing temperature and duration in the recovery period.

| Temperature (°C) | Time (h) | Fq′/Fm′ (BS)^⸸^ | SE^⸸⸸⸸^ | Fq′/Fv′  (BS) | SE | qL´  (BS) | SE | Fq′/Fm′ (24h AS)^⸸⸸^ | SE | Fq′/Fv′  (24h AS) | SE | qL´  (24h AS) | SE |
| --- | --- | --- | --- | --- | --- | --- | --- | --- | --- | --- | --- | --- | --- |
| 0 | 1 | 0.774 | 0.027 | 1.000 | 0.003 | 1.003 | 0.009 | 0.739a-c | 0.008 | 0.997 | 0.001 | 1.005 | 0.003 |
| 0 | 2 | 0.738 | 0.005 | 0.999 | 0.001 | 1.001 | 0.002 | 0.785a | 0.030 | 1.038 | 0.041 | 0.905 | 0.103 |
| 0 | 3 | 0.762 | 0.010 | 1.002 | 0.004 | 0.996 | 0.010 | 0.739a-c | 0.006 | 0.999 | 0.003 | 1.002 | 0.005 |
| 0 | 4 | 0.772 | 0.007 | 0.999 | 0.001 | 1.002 | 0.002 | 0.752a-c | 0.002 | 1.000 | 0.002 | 1.001 | 0.003 |
| 0 | 5 | 0.752 | 0.009 | 0.997 | 0.001 | 1.006 | 0.001 | 0.754ab | 0.008 | 0.999 | 0.004 | 1.002 | 0.007 |
| -6 | 1 | 0.774 | 0.027 | 1.000 | 0.003 | 1.003 | 0.009 | 0.744a-c | 0.006 | 0.997 | 0.002 | 1.006 | 0.004 |
| -6 | 2 | 0.738 | 0.005 | 0.999 | 0.001 | 1.001 | 0.002 | 0.759ab | 0.009 | 1.004 | 0.002 | 0.991 | 0.006 |
| -6 | 3 | 0.762 | 0.010 | 1.002 | 0.004 | 0.996 | 0.010 | 0.757ab | 0.007 | 1.001 | 0.003 | 0.998 | 0.007 |
| -6 | 4 | 0.772 | 0.007 | 0.999 | 0.001 | 1.002 | 0.002 | 0.751a-c | 0.011 | 0.999 | 0.001 | 1.003 | 0.003 |
| -6 | 5 | 0.752 | 0.009 | 0.997 | 0.001 | 1.006 | 0.001 | 0.752ab | 0.005 | 0.999 | 0.008 | 1.003 | 0.017 |
| -8 | 1 | 0.774 | 0.027 | 1.000 | 0.003 | 1.003 | 0.009 | 0.735a-c | 0.004 | 1.002 | 0.002 | 0.996 | 0.003 |
| -8 | 2 | 0.738 | 0.005 | 0.999 | 0.001 | 1.001 | 0.002 | 0.716a-d | 0.014 | 0.998 | 0.002 | 1.003 | 0.003 |
| -8 | 3 | 0.762 | 0.010 | 1.002 | 0.004 | 0.996 | 0.010 | 0.722a-d | 0.010 | 1.002 | 0.002 | 0.997 | 0.003 |
| -8 | 4 | 0.772 | 0.007 | 0.999 | 0.001 | 1.002 | 0.002 | 0.679a-d | 0.008 | 1.004 | 0.003 | 0.996 | 0.003 |
| -8 | 5 | 0.752 | 0.009 | 0.997 | 0.001 | 1.006 | 0.001 | 0.720a-d | 0.007 | 1.004 | 0.002 | 0.994 | 0.003 |
| -10 | 1 | 0.774 | 0.027 | 1.000 | 0.003 | 1.003 | 0.009 | 0.583de | 0.102 | 1.000 | 0.001 | 1.001 | 0.001 |
| -10 | 2 | 0.738 | 0.005 | 0.999 | 0.001 | 1.001 | 0.002 | 0.730 a-c | 0.017 | 0.996 | 0.003 | 1.008 | 0.006 |
| -10 | 3 | 0.762 | 0.010 | 1.002 | 0.004 | 0.996 | 0.010 | 0.621b-e | 0.098 | 0.991 | 0.007 | 0.998 | 0.004 |
| -10 | 4 | 0.772 | 0.007 | 0.999 | 0.001 | 1.002 | 0.002 | 0.611c-e | 0.090 | 0.991 | 0.005 | 1.002 | 0.007 |
| -10 | 5 | 0.752 | 0.009 | 0.997 | 0.001 | 1.006 | 0.001 | 0.497e | 0.064 | 0.990 | 0.005 | 1.000 | 0.002 |
| -12 | 1 | 0.774 | 0.027 | 1.000 | 0.003 | 1.003 | 0.009 | 0.658a-d | 0.011 | 1.004 | 0.002 | 0.997 | 0.002 |
| -12 | 2 | 0.738 | 0.005 | 0.999 | 0.001 | 1.001 | 0.002 | 0.692a-d | 0.007 | 1.000 | 0.002 | 1.001 | 0.002 |
| -12 | 3 | 0.762 | 0.010 | 1.002 | 0.004 | 0.996 | 0.010 | 0.508e | 0.111 | 0.989 | 0.007 | 0.998 | 0.007 |
| -12 | 4 | 0.772 | 0.007 | 0.999 | 0.001 | 1.002 | 0.002 | 0.492e | 0.114 | 0.987 | 0.015 | 0.988 | 0.011 |
| -12 | 5 | 0.752 | 0.009 | 0.997 | 0.001 | 1.006 | 0.001 | 0.145f | 0.058 | 1.011 | 0.024 | 1.007 | 0.021 |

^⸸^Columns without letters are statistically insignificant.

^⸸⸸^In each column, means with similar letters do not have a significant difference in the probability level of 0.05 based on the least significant difference (LSD) test.

^⸸⸸⸸^Standard error

Abbreviation: BS (before stress) and AS (after stress).

***Continue the table*** ***1S***

| Temperature (°C) | Time (h) | Fq′/Fm′  (72h AS) | SE | Fq′/Fv′  (72h AS) | SE | qL´  (72h AS) | SE | Fq′/Fm′  (120h AS) | SE | Fq′/Fv′  (120h AS) | SE | qL´  (120h AS) | SE |
| --- | --- | --- | --- | --- | --- | --- | --- | --- | --- | --- | --- | --- | --- |
| 0 | 1 | 0.701a | 0.009 | 1.002 | 0.001 | 0.998 | 0.002 | 0.733ab | 0.006 | 0.996a | 0.002 | 1.008a | 0.003 |
| 0 | 2 | 0.720a | 0.006 | 1.003 | 0.002 | 0.996 | 0.003 | 0.739ab | 0.011 | 1.002a | 0.002 | 0.996a | 0.004 |
| 0 | 3 | 0.719a | 0.007 | 1.007 | 0.003 | 0.989 | 0.005 | 0.726ab | 0.016 | 1.000a | 0.003 | 1.000a | 0.004 |
| 0 | 4 | 0.721a | 0.005 | 1.002 | 0.002 | 0.997 | 0.004 | 0.734ab | 0.009 | 1.002a | 0.002 | 0.997a | 0.003 |
| 0 | 5 | 0.724a | 0.009 | 0.998 | 0.003 | 1.003 | 0.005 | 0.711ab | 0.004 | 1.001a | 0.001 | 0.999a | 0.002 |
| -6 | 1 | 0.731a | 0.021 | 1.000 | 0.003 | 0.998 | 0.004 | 0.729ab | 0.004 | 1.002a | 0.002 | 0.997a | 0.003 |
| -6 | 2 | 0.740a | 0.002 | 1.000 | 0.002 | 1.000 | 0.005 | 0.718ab | 0.008 | 1.004a | 0.003 | 0.994a | 0.005 |
| -6 | 3 | 0.745a | 0.006 | 1.002 | 0.002 | 0.996 | 0.003 | 0.730ab | 0.012 | 1.005a | 0.004 | 0.991a | 0.008 |
| -6 | 4 | 0.754a | 0.010 | 1.002 | 0.004 | 0.997 | 0.008 | 0.737ab | 0.003 | 1.004a | 0.002 | 0.993a | 0.003 |
| -6 | 5 | 0.751a | 0.008 | 0.938 | 0.060 | 2.880 | 1.875 | 0.723ab | 0.010 | 0.996a | 0.003 | 1.006a | 0.004 |
| -8 | 1 | 0.751a | 0.014 | 1.008 | 0.007 | 0.981 | 0.017 | 0.798a | 0.005 | 1.002a | 0.004 | 0.994a | 0.012 |
| -8 | 2 | 0.746a | 0.002 | 0.943 | 0.060 | 2.622 | 1.625 | 0.792ab | 0.004 | 0.999a | 0.002 | 1.003a | 0.004 |
| -8 | 3 | 0.734a | 0.009 | 1.001 | 0.001 | 0.999 | 0.001 | 0.806a | 0.006 | 1.005a | 0.006 | 0.985a | 0.018 |
| -8 | 4 | 0.746a | 0.009 | 1.009 | 0.006 | 0.982 | 0.013 | 0.787ab | 0.009 | 0.999a | 0.001 | 1.004a | 0.002 |
| -8 | 5 | 0.735a | 0.006 | 0.999 | 0.003 | 1.003 | 0.005 | 0.787ab | 0.010 | 1.000a | 0.001 | 1.000a | 0.003 |
| -10 | 1 | 0.757a | 0.004 | 1.004 | 0.003 | 0.994 | 0.007 | 0.774ab | 0.002 | 0.999a | 0.001 | 1.002a | 0.004 |
| -10 | 2 | 0.766a | 0.005 | 1.000 | 0.002 | 0.999 | 0.005 | 0.771ab | 0.005 | 1.000a | 0.001 | 1.000a | 0.003 |
| -10 | 3 | 0.756a | 0.010 | 1.005 | 0.002 | 0.991 | 0.005 | 0.759ab | 0.013 | 0.999a | 0.002 | 1.002a | 0.004 |
| -10 | 4 | 0.765a | 0.004 | 1.006 | 0.002 | 0.987 | 0.005 | 0.777ab | 0.003 | 1.000a | 0.002 | 0.999a | 0.004 |
| -10 | 5 | 0.527b | 0.176 | 0.752 | 0.251 | 0.748 | 0.249 | 0.170c | 0.170 | 0.250b | 0.250 | 0.250b | 0.250 |
| -12 | 1 | 0.787a | 0.002 | 1.001 | 0.002 | 0.998 | 0.006 | 0.782ab | 0.003 | 1.003a | 0.004 | 0.994a | 0.010 |
| -12 | 2 | 0.783a | 0.005 | 1.003 | 0.002 | 0.993 | 0.006 | 0.770ab | 0.016 | 1.017a | 0.019 | 0.965a | 0.039 |
| -12 | 3 | 0.735a | 0.029 | 1.002 | 0.001 | 0.995 | 0.003 | 0.638b | 0.090 | 1.003a | 0.002 | 1.001a | 0.002 |
| -12 | 4 | 0.686a | 0.036 | 0.999 | 0.005 | 1.000 | 0.004 | 0.197c | 0.197 | 0.253b | 0.253 | 0.243b | 0.243 |
| -12 | 5 | 0.471b | 0.159 | 0.749 | 0.250 | 0.751 | 0.250 | 0.000d | 0.000 | 0.000c | 0.000 | 0.000c | 0.000 |
